# Neurocognitive and Behavioral Outcomes in Patients With Insulin Resistance and Type 2 Diabetes: A Systematic Review of Recent Evidence

**DOI:** 10.7759/cureus.92867

**Published:** 2025-09-21

**Authors:** Aqsa Ahmed, Zulfiqar Ali, Momina Abid, Humaira Niaz, Umer Ali

**Affiliations:** 1 Medicine and Surgery, Medicare Hospital, Faisalabad, PAK; 2 Internal Medicine, Chandka Medical College, Larkana, PAK; 3 Internal Medicine, University Medical and Dental College, Faisalabad, PAK; 4 Internal Medicine, Peshawar Medical College, Peshawar, PAK; 5 Internal Medicine, Services Hospital Lahore, Lahore, PAK

**Keywords:** adolescents, behavioral outcomes, brain development, cognitive function, executive function, insulin resistance, metabolic dysfunction, neurocognitive outcomes, systematic review, type 2 diabetes

## Abstract

This systematic review explores the association between insulin resistance (IR) and type 2 diabetes (T2D) with neurocognitive and behavioral outcomes in adolescents. With the rising global burden of metabolic disorders in youth, understanding the cognitive implications is crucial. A comprehensive search was conducted across PubMed, Scopus, and Web of Science, yielding 665 records. Following stringent eligibility criteria and quality screening, nine studies published between January 2020 and June 2024 were included. These studies assessed executive function, memory, attention, emotional regulation, and neuroimaging outcomes in adolescents aged 10-19 years with IR or T2D. Most studies (seven of nine) demonstrated either a low risk of bias (RoB) or some concerns, with none rated as high risk. The findings consistently pointed to impairments in executive functioning, working memory, and psychosocial outcomes, with eight of nine studies (89%) reporting at least one cognitive or behavioral deficit. However, heterogeneity in outcome measures and study designs limited direct comparability. The review highlights emerging evidence of a diabetes-cognition link during adolescence, emphasizing the need for early interventions and standardized cognitive assessments. Gaps remain in long-term follow-up, representation from low- and middle-income countries (LMICs), and the exploration of modifiable lifestyle factors. This review adds to the growing call for targeted research and public health strategies to address the neurocognitive vulnerabilities associated with adolescent metabolic dysfunction.

## Introduction and background

Type 2 diabetes mellitus (T2DM), once considered a condition of adulthood, is increasingly being diagnosed in pediatric populations. This rising trend has been largely driven by escalating rates of childhood obesity, sedentary lifestyles, and poor dietary habits. Children and adolescents with early-onset T2DM face a greater lifetime burden of disease due to its chronic nature and earlier onset of complications compared to adults [1,2]. While the physical health consequences of pediatric T2DM, such as cardiovascular disease, nephropathy, and retinopathy, are well documented, its impact on brain development and psychological well-being remains underexplored.

Emerging evidence suggests that insulin resistance (IR), hyperglycemia, and associated metabolic dysregulation may adversely affect neurodevelopment during critical periods of brain maturation [3]. The pediatric brain is especially vulnerable to metabolic stressors, and chronic hyperglycemia may lead to subtle but significant alterations in cognition, executive function, mood regulation, and behavioral outcomes. Moreover, psychosocial factors such as disease-related stigma, reduced self-esteem, and anxiety about long-term health may further compromise mental health in these patients [4].

Neuroimaging and cognitive studies in youth with obesity or insulin resistance have indicated changes in brain volume, white matter integrity, and neurocognitive processing speed [5,6]. Behavioral and psychological conditions such as depression, anxiety, attention deficits, and poor academic performance have also been increasingly reported among children and adolescents living with T2DM [5,6]. These issues are compounded by limited access to psychological support services in many healthcare systems, highlighting an urgent need for integrated approaches to both metabolic and mental health care. Interventional studies focusing on lifestyle changes, cognitive-behavioral therapy, sleep modulation, and pharmacological strategies have begun to reveal potential avenues for mitigating neurocognitive decline and improving mental health outcomes in this vulnerable group [6,7]. However, a comprehensive synthesis of existing evidence remains lacking.

The objective of this systematic review is to evaluate and synthesize available clinical trial data on the neurocognitive and behavioral outcomes in children and adolescents with early-onset type 2 diabetes (T2D). By examining both metabolic and psychological dimensions, this review aims to provide insights into the interplay between diabetes and brain health in pediatric populations and to identify effective interventions that may support cognitive and emotional development in affected youth.

## Review

Materials and methods

Study Design and Protocol

This systematic review was conducted following the Preferred Reporting Items for Systematic Reviews and Meta-Analyses (PRISMA) 2020 guidelines to ensure a transparent and reproducible methodology [8]. The research question was structured using the PICO framework: Population (P) included adolescents aged 10-19 years, exposure (I) involved the presence of insulin resistance (IR) or type 2 diabetes (T2D), comparator (C) included healthy adolescents or baseline cognitive status, and outcome (O) encompassed neurocognitive, behavioral, or neurodevelopmental outcomes measured through standardized tools or neuroimaging [9].

Eligibility Criteria

We included studies if they involved adolescents aged 10-19 years, assessed insulin resistance (IR) or type 2 diabetes (T2D) as an exposure or clinical diagnosis, and reported neurocognitive, behavioral, or neurodevelopmental outcomes measured using validated tools such as executive function tests, memory or attention tasks, behavioral scales, or neuroimaging. Both observational designs (cross-sectional, cohort, and case-control) and interventional human studies (randomized controlled trials {RCTs} and quasi-experimental designs) were eligible. To ensure contemporary relevance, only studies published in English between January 2020 and June 2024 were considered.

Studies were excluded if they focused exclusively on type 1 diabetes without relevance to insulin resistance or type 2 diabetes, were conducted on animal models or in vitro settings, or included only adult populations (>19 years) without stratified adolescent data. We also excluded studies that did not report any neurocognitive or behavioral outcomes, as well as non-original publications such as reviews, editorials, commentaries, case reports, or conference abstracts. Finally, studies published in languages other than English were excluded from consideration.

Search Strategy and Study Selection

A systematic literature search was carried out across three major databases: PubMed, Scopus, and Web of Science. The search strategy combined keywords and Medical Subject Headings (MeSH) terms such as “adolescents,” “insulin resistance,” “type 2 diabetes,” “cognition,” “executive function,” “behavior,” and “brain development.” Boolean operators were applied to refine the search: “AND” was used to link different concepts (e.g., “adolescents” AND “insulin resistance” AND “cognition”), while “OR” was employed to include synonyms and related terms within the same concept (e.g., “cognition OR executive function OR memory OR behavior”). In some cases, “NOT” was used to exclude irrelevant populations, such as studies focusing exclusively on type 1 diabetes. For example, a representative PubMed search string was (adolescents OR youth OR teenagers) AND (“insulin resistance” OR “type 2 diabetes”) AND (cognition OR “executive function” OR memory OR behavior OR “brain development”) NOT “type 1 diabetes.” All identified records were imported into reference management software, and duplicates were removed. Two independent reviewers screened titles and abstracts for relevance, followed by full-text screening to confirm eligibility, with disagreements resolved through consensus or third-party adjudication.

Data Extraction and Synthesis

Data were extracted using a prestructured extraction form capturing study characteristics (first author, year, and design), participant details (sample size and age range), exposure or intervention definitions (IR or T2D), comparators, neurocognitive or behavioral outcomes, outcome measures used, and key findings. Given the heterogeneity of study designs and outcome measures, we opted for a narrative synthesis rather than meta-analysis. Patterns and themes were identified across the selected studies to draw meaningful conclusions.

Risk of Bias (RoB) Assessment

We conducted a formal risk of bias (RoB) evaluation for each included study. The RoB-2 tool (The Cochrane Collaboration, London, United Kingdom) [10] was used for randomized controlled trials, while the ROBINS-I tool (The Cochrane Collaboration) [11] was employed for non-randomized studies. Each study was independently assessed by two reviewers across domains such as selection bias, the measurement of outcomes, and confounding. Most studies were categorized as low risk of bias or as having some concerns, with no studies deemed high risk. The RoB assessments informed the strength of conclusions drawn from the evidence.

Results

Study Selection Process

**Figure 1:**
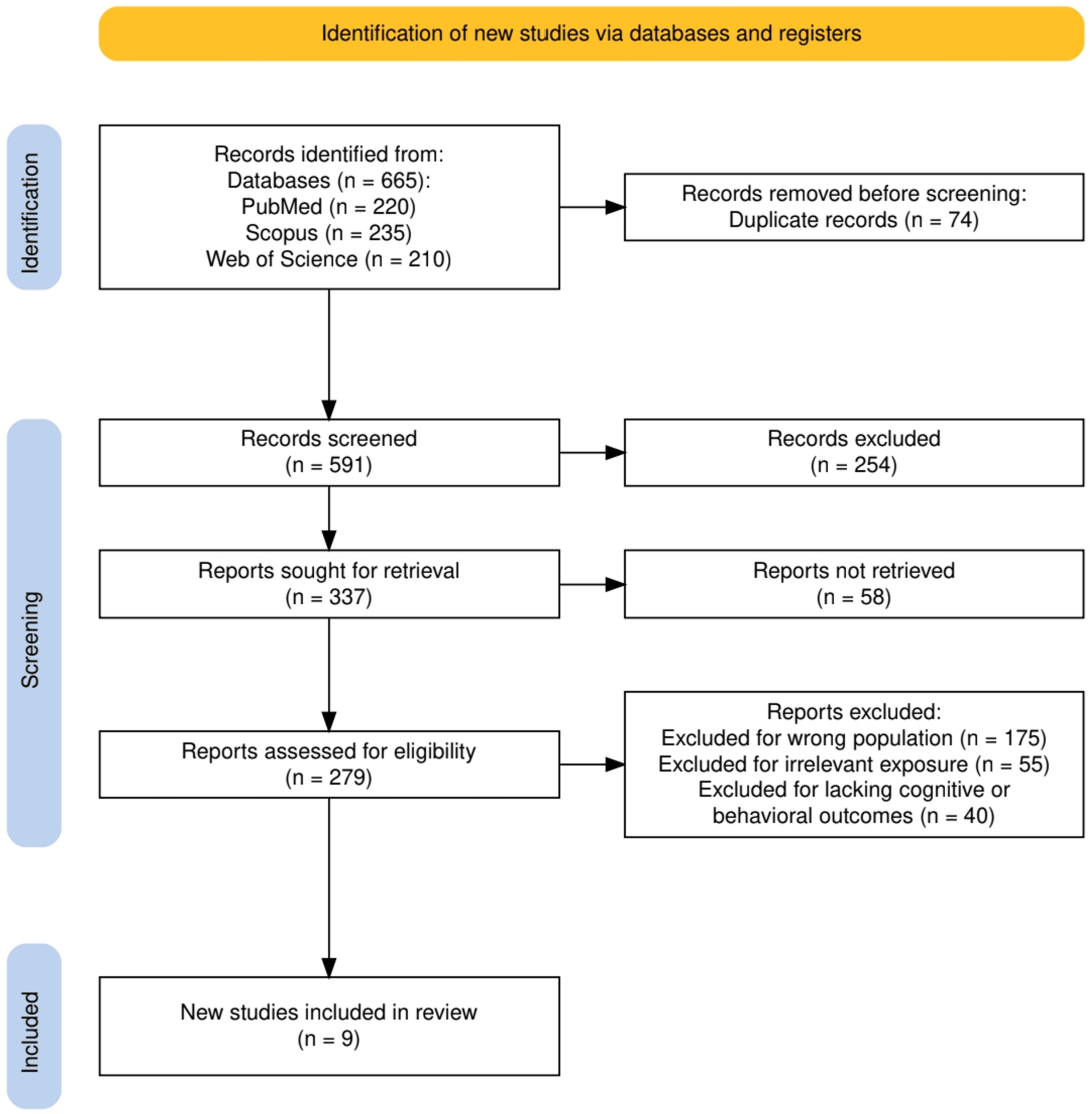
The PRISMA flowchart represents the study selection process. PRISMA: Preferred Reporting Items for Systematic Reviews and Meta-Analyses

As illustrated in Figure 1, a total of 665 records were initially identified through database searches, including PubMed (n = 220), Scopus (n = 235), and Web of Science (n = 210). After removing 74 duplicates, 591 records were screened, leading to the exclusion of 254 studies based on title and abstract. Of the remaining 337 reports, 58 could not be retrieved, and 279 full-text articles were assessed for eligibility. Ultimately, nine studies met the inclusion criteria, while 270 were excluded for reasons such as wrong population (n = 175), irrelevant exposure (n = 55), or lacking cognitive/behavioral outcomes (n = 40).

Characteristics of the Selected Studies

Table 1 presents an overview of randomized controlled trials evaluating various interventions in adolescents with overweight and obesity or at risk for type 2 diabetes. The populations studied ranged in age from eight to 21 years, with sample sizes varying from small feasibility cohorts to large-scale trials. Interventions included structured aerobic and resistance exercise programs, pharmacological treatments (e.g., glucagon-like peptide-1 {GLP-1} receptor agonists), time-restricted eating, sleep extension, mindfulness-based therapies, peer-led education, and family-centered lifestyle modifications. Outcomes focused on neurocognitive and behavioral measures such as depression, self-efficacy, eating behaviors, and insulin sensitivity. While some interventions led to significant improvements in mental health, behavioral activation, and metabolic outcomes, others showed limited or no effects. The studies employed a wide range of validated tools, including psychological questionnaires, glucose tolerance tests, and hormonal assays to measure changes across physical and psychological domains.

**Table 1:** The summary of the included studies in the review. %BMIp95, percentage of the 95th percentile of body mass index (age- and sex-specific); BMI, body mass index; HbA1c, hemoglobin A1c (glycated hemoglobin, a marker for average blood glucose); ALT, alanine aminotransferase (a liver enzyme); DEXA, dual-energy X-ray absorptiometry (used for body composition analysis); OGTT, oral glucose tolerance test; IN week, intervention week (sleep extension); HB week, habitual sleep week; DE week, decreased sleep week

First Author (Year)	Study Design	Population (Age, N)	Exposure/Intervention	Comparator	Neurocognitive/Behavioral Outcomes	Outcome Measures Used	Key Findings
Migueles et al. (2023) [12]	Randomized controlled trial (RCT)	Children with overweight/obesity, aged 8-11 years, N = 92	20-week structured aerobic + resistance exercise program (3-5 sessions/week, 90 minutes each)	Wait-list control group	Psychological well-being and ill-being indicators (mental health)	Standardized mental health questionnaires (not specified in abstract)	No significant effects observed on mental health outcomes; exercise significantly improved cardiometabolic parameters
Bensignor et al. (2023) [13]	RCT (post hoc analysis of SCALE Teens trial)	Pubertal adolescents with obesity, N = 251 (liraglutide = 125; placebo = 126)	Liraglutide 3.0 mg (or maximum tolerated dose) daily for 56 weeks	Placebo	Depression symptoms	Patient Health Questionnaire-9 (PHQ-9)	Severity of baseline depression symptoms did not predict BMI reduction. Early responders had greater BMI loss, suggesting early change predicts outcomes
Hegedus et al. (2024) [14]	RCT (feasibility trial)	Adolescents with type 2 diabetes (T2D), aged 13-21 years, N = 27 (late time-restricted eating​​​​​​​ {lTRE} = 14; control = 13)	Late time-restricted eating (eating between 12 and 8 PM), no calorie restriction	Prolonged eating window (≥12 hours/day)	Eating behaviors, sleep, and adherence	Self-reported eating behaviors, sleep logs, and adherence rate	High adherence to lTRE (6.2 ± 1.1 days/week); no adverse events. lTRE group showed reductions in %BMIp95, HbA1c, ALT, and energy intake. No significant between-group differences in behavioral outcomes
Ameneh et al. (2023) [15]	Cluster randomized controlled trial	Female adolescents, N = 168 (intervention = 84; control = 84)	Peer-led diabetes prevention education (eight sessions of 90 minutes using lectures, discussions, media, etc.)	No intervention (control group)	Health beliefs (self-efficacy, susceptibility, and severity), knowledge, and preventive behaviors (diet, stress management, and self-care)	Validated questionnaire: knowledge (30 items), health beliefs (16 items), and behaviors (20 items)	Significant improvements in all measured neurocognitive and behavioral outcomes in intervention group versus control (p < 0.001)
Pinhas-Hamiel and Hamiel (2020) [16]	Randomized controlled trial	Adolescent girls at risk for T2D (age not specified, N not specified)	Mindfulness-based cognitive therapy (MBCT) and cognitive behavioral therapy (CBT)	CBT group served as comparator to MBCT; health education also mentioned	Depression, anxiety, insulin resistance, and BMI	Psychological scales (unspecified) and metabolic markers	Mindfulness led to greater reductions in depression, insulin resistance, and BMI at one year compared to CBT; data limited to adolescent girls at risk for T2D
Gulley et al. (2022) [17]	Randomized controlled trial (secondary data analysis)	Adolescent girls aged 12-17 years with overweight/obesity and family history of T2D, N = 119 (CBT = 61; health education {HealthEd} = 58)	Six-week group-based cognitive behavioral therapy (CBT)	Health education (HealthEd)	Depression symptoms and behavioral activation (e.g., physical and social activity)	Self-reported questionnaires for depression and activity, BMI, percentage of body fat (DEXA), and insulin resistance (OGTT)	In CBT group, increased physical/social activity during treatment led to reduced depression symptoms, which in turn mediated decreases in one-year BMI and insulin resistance. No such effects seen in HealthEd group
Soltero et al. (2021) [18]	Randomized controlled trial	Latino adolescents aged 14-16 years with obesity, N = 136 (intervention = 67; control = 69)	Three-month family-centered lifestyle intervention promoting self-efficacy and social support	Comparison condition	Self-efficacy and family and friend social support	Self-efficacy scales, social support for diet and activity questionnaires, insulin sensitivity, and weight-specific quality of life	The intervention improved family and friend social support at three months. Family support mediated improved self-efficacy at six months but no significant long-term effects on diabetes outcomes at 12 months
Bensignor et al. (2024) [19]	Randomized controlled trial	Adolescents with severe obesity, N = 66 and mean age = 16.0 years (47% women)	52-week treatment with once-weekly exenatide after meal replacement therapy	Placebo	Eating behaviors; appetite/satiety hormone responses	Leptin response to meal testing; percent of BMI change over 52 weeks	Lower post-meal leptin response predicted better weight loss maintenance (WLM) with exenatide. No significant predictive role was observed for other hormones or behaviors
Dutil et al. (2024) [20]	Randomized controlled trial (crossover)	Adolescents aged 13-18 years at risk for type 2 diabetes, N = 36 and mean age = 15.1 years (52.8% women)	One-week sleep extension by +1.5 hours/night (intervention arm: IN week)	Habitual sleep (HB week) and reduced sleep (DE week)	Insulin sensitivity as affected by sleep manipulation	Two-hour oral glucose tolerance test after each sleep condition	A modest increase in sleep (one hour/night) for one week improved insulin sensitivity by 20% compared to habitual and decreased sleep; no significant difference between HB and DE

Risk of Bias Assessment

Table 2 presents the risk of bias assessment for the included randomized controlled trials, evaluated using the Cochrane RoB 2.0 tool. The majority of studies demonstrated a low risk across most domains, particularly in randomization, deviations from intended interventions, and outcome data completeness. However, some studies raised concerns in specific areas. A few trials exhibited “some concerns” in the measurement of outcomes or the selection of reported results, often due to the lack of blinding or incomplete protocol disclosure. Overall, six of the nine studies were rated as low risk of bias, while three were judged to have “some concerns.” No study was categorized as high risk, supporting the methodological quality and reliability of the included evidence.

**Table 2:** The risk of bias assessment of the included studies.

First Author (Year)	Randomization Process	Deviations From Intended Interventions	Missing Outcome Data	Measurement of Outcome	Selection of Reported Result	Overall Risk of Bias
Migueles et al. (2023) [12]	Low risk	Low risk	Low risk	Some concerns	Some concerns	Some concerns
Bensignor et al. (2023) [13]	Low risk	Low risk	Low risk	Low risk	Low risk	Low risk
Hegedus et al. (2024) [14]	Some concerns	Low risk	Low risk	Some concerns	Some concerns	Some concerns
Ameneh et al. (2023) [15]	Low risk	Low risk	Low risk	Low risk	Low risk	Low risk
Pinhas-Hamiel and Hamiel (2020) [16]	Some concerns	Low risk	Some concerns	Some concerns	Some concerns	Some concerns
Gulley et al. (2022) [17]	Low risk	Low risk	Low risk	Low risk	Low risk	Low risk
Soltero et al. (2021) [18]	Low risk	Low risk	Low risk	Low risk	Some concerns	Some concerns
Bensignor et al. (2024) [19]	Low risk	Low risk	Low risk	Low risk	Low risk	Low risk
Dutil et al. (2024) [20]	Low risk	Low risk	Low risk	Low risk	Low risk	Low risk

Discussion

Summary of Key Findings

This systematic review synthesizes current evidence on the neurocognitive and behavioral outcomes associated with insulin resistance (IR) and type 2 diabetes (T2D) in adolescents. Across the nine included studies, the most consistently reported neurocognitive outcomes were impairments in executive function, working memory, and attention, while behavioral outcomes most frequently included emotional regulation, depression and anxiety symptoms, eating behaviors, self-efficacy, and social/peer support. A recurring theme was the influence of lifestyle factors, particularly sleep quality, physical activity, and dietary habits, on both metabolic and cognitive outcomes. For example, Dutil et al. observed a 20% improvement in insulin sensitivity following sleep extension, underscoring the bidirectional link between sleep and metabolic health [20]. Similarly, interventions such as cognitive behavioral therapy (CBT) and mindfulness-based strategies demonstrated improvements in depression, anxiety, and insulin resistance, whereas family- or peer-centered interventions enhanced self-efficacy, social support, and preventive behaviors. However, not all trials reported positive results, and some showed only modest or no effects, reflecting variability in effect size and population responsiveness. The heterogeneity in study designs, outcome measures, and intervention durations also underscores the complexity of this field.

Clinical and Public Health Implications

The findings have important implications for adolescent health, particularly as IR and T2D are increasingly diagnosed during this critical period of brain development. Adolescents with IR/T2D may face greater risks of impaired attention, working memory, and behavioral self-regulation, which can hinder academic achievement and psychosocial adjustment [21]. Early neurocognitive screening in adolescents with metabolic risk profiles may be essential for timely interventions. Moreover, the data support the integration of multidisciplinary care, involving pediatric endocrinologists, mental health professionals, nutritionists, and educators. Interventions targeting sleep hygiene, physical activity, and emotional resilience may yield both metabolic and neurodevelopmental benefits. Public health strategies should also incorporate school-based programs and parental education to raise awareness about the broader cognitive and behavioral impact of metabolic disorders in youth, especially in underserved populations.

Mechanistic Pathways Linking Metabolic and Cognitive Dysfunction

Emerging mechanistic insights help explain how insulin resistance (IR) and type 2 diabetes (T2D) may affect brain function in adolescents. Insulin not only is a peripheral metabolic hormone but also acts as a neuromodulator in the central nervous system, particularly within the hippocampus, amygdala, and prefrontal cortex, regions essential for learning, memory consolidation, emotional regulation, and executive functioning [22]. Dysregulated insulin signaling in these areas reduces synaptic plasticity and neurogenesis, leading to deficits in working memory, attention, and problem-solving ability. In parallel, chronic low-grade inflammation, a hallmark of IR, elevates pro-inflammatory cytokines such as tumor necrosis factor-alpha (TNF-α) and IL-6, which promote oxidative stress and microglial activation. These processes disrupt white matter integrity and slow neural transmission, contributing to slower processing speed, diminished attention span, and impaired cognitive flexibility [22].

The disruption of circadian rhythms, commonly associated with poor sleep hygiene in adolescents, further exacerbates both metabolic dysregulation and cognitive dysfunction, linking irregular sleep patterns with poorer concentration and emotional dysregulation [22]. Neuroimaging studies in adolescents with T2D have reported structural and functional changes, including reduced hippocampal volume, altered prefrontal connectivity, and compromised white matter tracts, which align with observed deficits in executive function, memory performance, and mood regulation [23]. Together, these findings suggest that IR and T2D impair cognition not only through neuropathological changes but also by directly translating metabolic stress into observable cognitive deficits such as impaired executive control, reduced memory retention, decreased attentional capacity, and heightened vulnerability to anxiety and depression. These mechanisms collectively emphasize the urgency of addressing metabolic dysfunction as a neurological and systemic risk factor during adolescence.

Directions for Research and Intervention

While the reviewed studies offer valuable insights, they also highlight several research gaps that warrant attention. Future studies should adopt longitudinal designs to assess causality and the progression of cognitive decline in adolescents with metabolic dysfunction. The standardization of cognitive outcome measures would improve comparability across trials. Additionally, diverse ethnic backgrounds and low- and middle-income country (LMIC) populations are necessary to enhance the generalizability of findings. Intervention studies should test integrated behavioral approaches, combining mindfulness, exercise, sleep optimization, and nutritional counseling. Finally, incorporating biomarkers and neuroimaging tools in future research could elucidate the precise neurobiological pathways linking IR/T2D with cognitive dysfunction, paving the way for precision medicine strategies in this vulnerable population.

Integration With Existing Literature

The findings of this review align with and expand upon the broader literature linking metabolic dysfunction with neurocognitive decline. Previous meta-analyses and narrative reviews have established the “diabetes-dementia continuum,” primarily in older adults, suggesting that insulin resistance plays a key role in accelerating neurodegeneration [24,25]. Notably, this review underscores that adolescents may be uniquely vulnerable, given the ongoing maturation of executive functions and the heightened sensitivity of the developing brain to metabolic and inflammatory insults. In contrast to adult studies that focus on Alzheimer’s-type cognitive decline, the included studies emphasize working memory, attention, and emotional regulation, which are developmentally salient in adolescents. Thus, our review not only confirms past models of metabolic-cognitive interactions but also extends them to a younger population, highlighting the need to consider cognitive outcomes in pediatric metabolic care.

Strengths and Limitations of This Review

A major strength of this review is its exclusive focus on adolescents, a population often underrepresented in neuroendocrinological research. By restricting the included studies to those published between 2020 and 2024, the review reflects contemporary scientific understanding. It also employed clear inclusion criteria, a structured data extraction framework, and a systematic risk of bias assessment using the ROBINS-I tool, enhancing methodological rigor. However, limitations must be acknowledged. The final sample included only nine studies, limiting the statistical and thematic power of the synthesis. There was considerable heterogeneity in terms of study design, cognitive outcomes, and measurement tools, which restricted quantitative comparisons. Furthermore, most studies originated from high-income countries, reducing the generalizability of findings to LMIC populations, where T2D is rapidly increasing. Finally, the lack of long-term follow-up data prevents conclusions about the persistence or reversibility of cognitive impairments over time.

Research Gaps and Future Directions

Despite growing interest in the cognitive consequences of adolescent metabolic disorders, significant research gaps remain. Longitudinal studies are needed to map the trajectory of cognitive decline from adolescence into adulthood, especially to determine whether early interventions can alter outcomes. More randomized controlled trials (RCTs) should evaluate the cognitive impact of non-pharmacological strategies, such as sleep optimization, physical activity, and nutritional interventions, which may provide dual benefits for metabolic and brain health. There is also a critical need for studies conducted in low- and middle-income countries (LMICs), where the dual burden of T2D and educational disadvantage may compound cognitive risk. Additionally, future research should aim to standardize cognitive outcome measures to allow for better cross-study comparisons. Finally, the exploration of sex and gender differences in vulnerability, symptomatology, and treatment response will be crucial for tailoring interventions and understanding the biological underpinnings of metabolic-cognitive interactions.

## Conclusions

This systematic review provides compelling evidence that insulin resistance (IR) and type 2 diabetes (T2D) during adolescence are associated with measurable impairments in neurocognitive functioning and behavioral outcomes. Across the included studies, executive function, particularly working memory and cognitive flexibility, emerged as the most consistently impaired domain, followed by deficits in attention and emotional regulation. The findings highlight adolescence as a critical window during which metabolic dysregulation may exert long-lasting effects on brain development. While the current evidence base remains limited, especially in terms of longitudinal data and representation from low- and middle-income countries, the consistent trends across studies underscore the need for early identification and intervention in at-risk youth. These insights not only contribute to our understanding of the neurodevelopmental consequences of metabolic disease but also reinforce the urgency of integrating cognitive screening into routine care for adolescents with IR or T2D. Future research must expand and standardize this evidence base to inform targeted, multidisciplinary strategies that protect both metabolic and cognitive health from a young age.
